# Diagnosis and Treatment Challenges of *Candida guilliermondii* in Immunocompromised Patients: A Case Study in a Neutropenic AML Patient

**DOI:** 10.1155/2024/7806235

**Published:** 2024-07-22

**Authors:** Dhruvi Modi, Sophie Dessureault, John Greene

**Affiliations:** ^1^ Gujarat Adani Institute of Medical Sciences, Bhuj, Gujarat, India; ^2^ GI Tumor Program Moffitt Cancer Center and Department of Oncologic Sciences University of South Florida Morsani College of Medicine, Tampa, Florida, USA; ^3^ Division of Infectious Diseases and Tropical Medicine Internal Medicine Department at Moffitt Cancer Center, Tampa, Florida, USA

## Abstract

Although fungal infections causing intestinal perforation and necrosis are rare, they can be particularly dangerous in immunosuppressed patients, often leading to increased mortality rates and poor prognoses. Candida species are typically surface fungi, but in patients with compromised immune systems, they can invade the small intestine and cause angioinvasive infections. A case study involving a 30-year-old female with acute myeloid leukemia (AML) illustrates this phenomenon. The patient was presented with symptoms of abdominal pain, fever, diarrhea, recurrent episodes of intestinal necrosis, hematomas due to thrombocytopenia, and subsequent postoperative enterocutaneous fistulas. Extensive testing ruled out other possible causes of intestinal necrosis and enteritis, including Crohn's and CMV diseases. *Candida guilliermondi* was ultimately identified in blood cultures from the periphery, peritoneal fluid, and intestinal biopsy of respected sections, indicating that it was responsible for intestinal invasion and necrosis. The patient was then treated with amphotericin B, cefepime, and metronidazole. This case highlights the potential severity of fungal infections in immunosuppressed patients, particularly Candida species, and the importance of prompt diagnosis and appropriate treatment.

## 1. Introduction

The *Candida guilliermondii* complex is a taxonomically complex group consisting of several morphologically indistinguishable species such as *C. guilliermondii*, *Candida fermentati*, *Candida carpophila*, and *Candida xestobii* [1]. After recent taxonomic revisions, the teleomorph (sexual reproductive form) of this yeast has been renamed *Meyerozyma guilliermondii*. However, infections are commonly caused by the anamorph (asexual reproductive form), which continues to be known as *Candida guilliermondii* [2].

*C. guilliermondii* is a relatively rare fungal yeast pathogen that usually acts as a saprophyte in humans. It can lead to serious infections in immunocompromised individuals, especially in those with hematological malignancies. This yeast has been isolated from a variety of sources, including human skin and mucosal surfaces, soil, insects, plants, seawater, and tree secretions [3]. This species is characterized by its production of only pseudohyphae and its ability to grow on Sabouraud dextrose agar (SDA), where it forms small, flat, or smooth colonies that are yellowish or creamish in color, along with short pseudohyphae [4].

In recent years, the number of invasive infections caused by yeasts has increased, and the *C. guilliermondii* complex accounts for 3.7% of all fungal infections in Latin America [5]. Additionally, previous studies have highlighted the reduced susceptibility of *C. guilliermondii* complex species to common antifungal agents, such as azoles and echinocandins [6].

## 2. Case Presentation

A 30-year-old female with a history of arthritis, HPV, irritable bowel syndrome, and COVID-19 was admitted to our institute on November 12, 2021, for induction chemotherapy (7 + 3 regimen with cytarabine, daunorubicin, gemtuzumab, and leuprolide) following a diagnosis of AML. The patient developed neutropenic fever and streptococcus mitis infection, which resolved.

**Figure 1:**
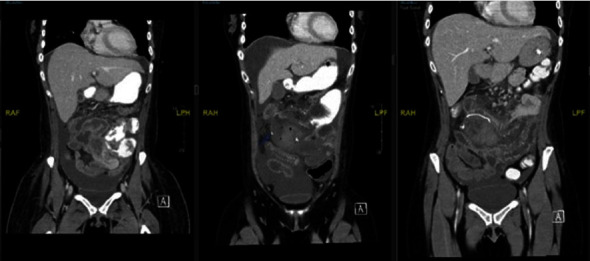
Necrosis of the small bowel. Initially reported as typhlitis but later reported as multiple segments of small bowel ischemia, possible anastomotic breakdown, small bowel obstruction, and enterocolitis. The CT scan shows an ischemic jejunum segment and enterocolitis.

**Figure 2:**
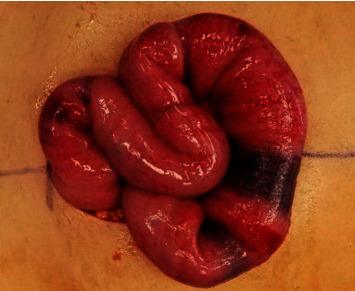
The ischemic segment (3 cm long) of the proximal jejunum, associated with mesenteric hematomas at the base of the ischemic segment.

Thirteen days after the chemotherapy, the patient presented with worsening abdominal pain, nausea, and vomiting. Physical examination revealed a soft, nondistended abdomen with significant periumbilical, rebound, and guarding tenderness. Laboratory tests showed neutropenia, with a white blood cell count of 0.3 cells/*μ*L and a platelet count of 7 platelets/*μ*L. Clinical symptoms suggestive of ischemia prompted an immediate abdominal CT scan, revealing an ischemic jejunal segment and enterocolitis (Figure 1). GI surgery was performed, and the patient underwent exploratory laparotomy. Operative findings included a 3 cm ischemic segment of the proximal jejunum associated with mesenteric hematomas (Figure 2). A 10 cm segment on either side was resected because of vascular compromise and impending perforation. The underlying etiology was attributed to thrombocytopenia, which led to intestinal and peri-intestinal hematoma.

**Figure 3:**
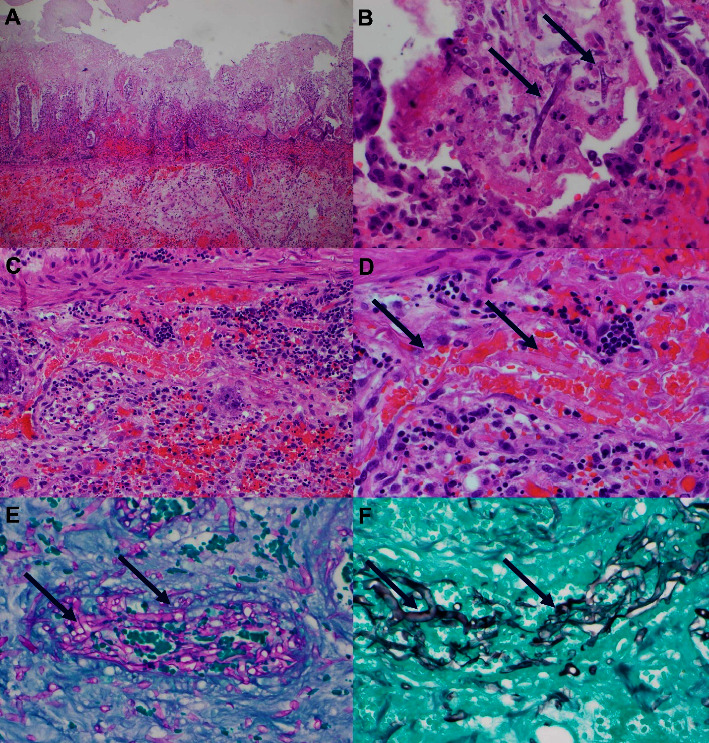
(A, B) Small intestine tissue with extensive mucosal injury, denudation, necrosis, and sloughed glandular epithelium. Note the fungal forms within the inflammatory-necrotic debris on surface (arrows). 40x (A) and 400x (B) magnification, respectively. (C, D) Congested and partially thrombosed vessels in the small intestine submucosa; fungal forms (arrows) were present in the vascular spaces intermixed with red blood cells. 100x (C) and 400x (D) magnification, respectively. (E, F) Fungal forms (arrows) are highlighted by periodic acid-Schiff stain ((E) 400x) and Grocott methenamine silver ((F) 400x) special stains, respectively.

**Figure 4:**
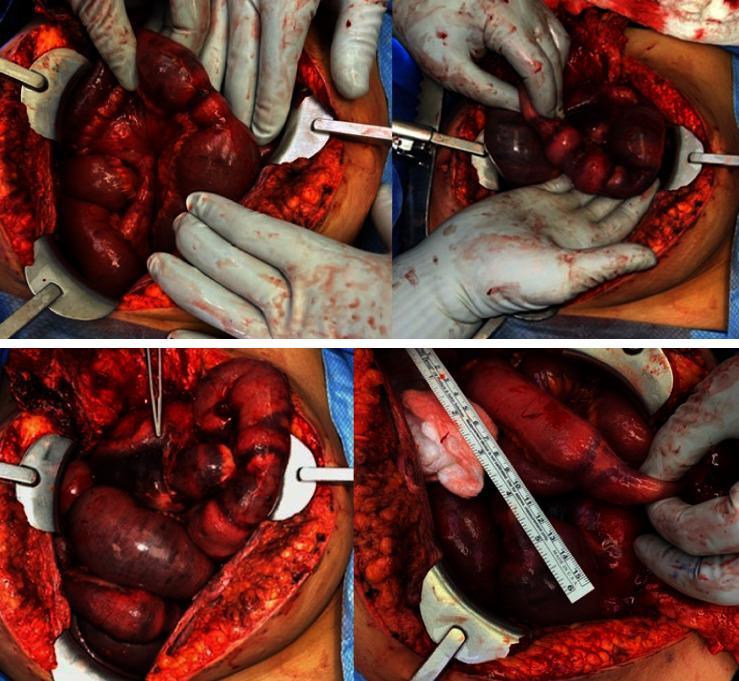
During surgical exploration, 13 skip areas of compromised small bowel were found, characterized by combinations of stenosis, hematomas, and ischemia. This figure shows stenosed segments of jejunum and necroses of the small intestine.

The respected specimens were sent for pathological examination, along with peripheral blood cultures. Pathology revealed necrosis and yeast in the vascular bed, and the peripheral blood cultures tested positive for *C. guilliermondii* (Figure 3). Zosyn and micafungin were initiated. Despite receiving antibiotics and her initial postoperative improvement, her condition declined after two days. The patient's pain escalated, and a repeat CT was warranted, which demonstrated an edematous small bowel wall proximal and distal to the anastomosis and thickening of the colon, suggesting enterocolitis, with the presence of increased peritoneal fluid with layering hemoperitoneum in the pelvis. The patient was brought back for a repeat surgery on December 1, 2021. Thirteen areas of bowel compromise, characterized by stenosis, hematomas, and ischemia, were found (Figure 4). The patient underwent five partial small bowel resections and hand-sewn end-to-end anastomoses. Cultures from November 30, 2021 to December 2, 2021 remained positive for *C. guilliermondii.* Fungal culture with a smear of the wound and peritoneal fluid confirmed the presence to be *Candida guillermondii*.

The patient was transitioned to amphotericin B liposomal 300 mg, 75 ml, 112.5 ml/hr, IVPB, Q24HR; cefepime 2000 mg, 16.67 ml/hr, IVPB, Q8HR; and metronidazole 500 mg, 100 ml, 200 ml/hr, IVPB, Q12HR.

**Figure 5:**
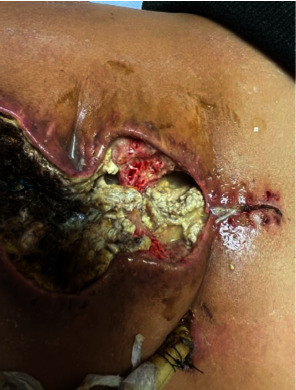
Abdominal wound dehiscence, fat necrosis, from the enterocutaneous fistula. The wound is infected with bacteria Enterobacter cloacae.

**Figure 6:**
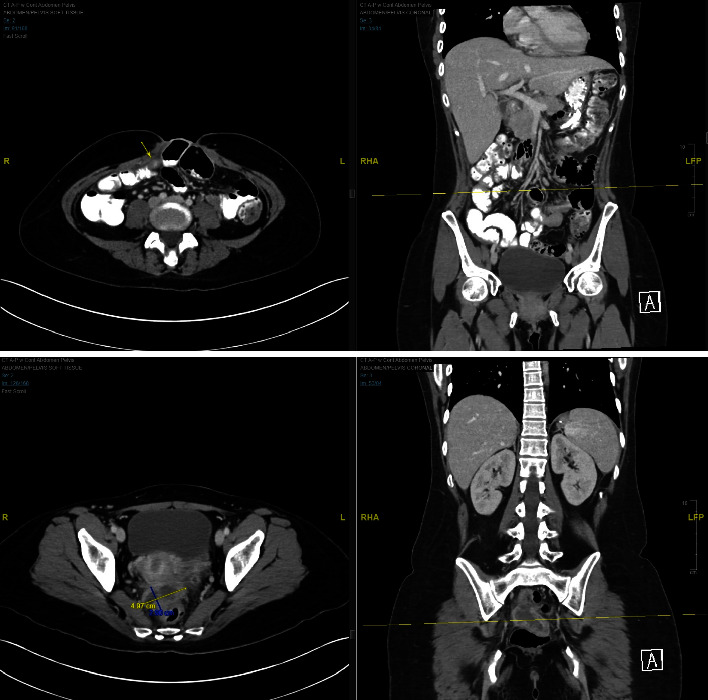
Persistent areas of wall thickening, consistent with enteritis; some areas appear to be slightly improved from the prior study. Layering hemoperitoneum in the pelvis seen in abdomen/pelvis soft view.

The patient developed partial wound dehiscence (Figure 5) (cultures positive for *Enterobacter cloacae*) and drainage of bilious fluid. A computed tomography (CT) scan on December 20, 2021 revealed an enterocutaneous fistula and loculated pockets of fluid within the abdomen and pelvis (Figure 6). The patient would not tolerate wound care at the bedside, so she was taken to the operating room on December 21, 2021 for wound debridement and catheter placement for fistula output control. The enterocutaneous fistula was managed with bowel rest, total parenteral nutrition, and drainage catheters. This allowed the patient to undergo three cycles of consolidation chemotherapy (March 4, 30, and April 28, 2022). During this period, the wound around the fistula healed. On June 17, 2022, the patient underwent takedown of the enterocutaneous fistula and ventral hernia repair. All subsequent scans and bone marrow biopsies revealed complete remission. Some of the peritoneal fluid from the perforation settled in the lower pelvis and formed a complex heterogeneous collection that was never removed but resolved in time. The patient was discharged on posaconazole (Table 1).

## 3. Discussion

This case highlights the importance of the early identification and management of fungal infections in patients with AML undergoing induction chemotherapy. In this case, we observed a 30-year-old female patient with Acute Myeloid Leukemia (AML) undergoing chemotherapy who developed a rare *Candida guilliermondii* infection that led to necrosis of the bowel wall and peritonitis. A critical contributor to the patient's vulnerability to *Candida guilliermondii* infection was neutropenia, which can be attributed to both AML and chemotherapy regimen. It is well established that neutropenia significantly diminishes the body's ability to fend off infections, and in this context, provides a favorable milieu for *Candida guilliermondii* to thrive. Our patient had neutropenic enterocolitis, which was identified by the presence of acute abdominal symptoms, neutropenia, and bowel wall thickening, which was evident from several radiological tests. The pathophysiology behind this type of invasive fungal infection is believed to be due to direct cytotoxicity on the intestinal wall caused by chemotherapy, followed by neutropenia and thrombocytopenia, which lead to spontaneous intramural hemorrhage or microvascular thrombosis [7].

Systemic candidiasis is a notable complication among patients with neutropenia and those undergoing cancer therapy [8]. The incidence of this infection has been on a consistent upward trajectory over the last 30 years and now constitutes a major cause of morbidity and mortality in high-risk demographics [9]. In the reviewed articles, the mortality rates range of 3.4–66.6%. In this regard, this infection had mortality rates of 11.76–66.6%, 13.6–54%, 16.66–18.8%, 59.25%, and 3.4% in Japan, Spain, Taiwan, United States, and Italy, respectively [10].

The factors that render neutropenic patients with hematological malignancies susceptible to systemic candidiasis vary depending on the extent of immune suppression and intricacies of the underlying malignancy.

It is important to recognize the digestive tract as a critical entry point for Candida species, especially in patients with acute neutropenia and leukemia [11, 12]. Being rich in endogenous microflora, the digestive tract is vulnerable to Candida ingress into the bloodstream through compromised anatomical barriers. The manifestations of Candida infections vary, including oropharyngeal candidiasis, esophagitis, candidemia, and acute or chronic disseminated candidiasis [4, 12].

In our patient, the proliferation of the Candida fungus can be attributed to her neutropenic state, which was compounded by the chemotherapy regimen. The therapy was instrumental in the breakdown of the intestinal mucosal barriers, paving the way for seeding of the fungus. However, a striking aspect of this case was the identification of *Candida guilliermondii* as the likely cause of necrosis in the bowel segments.

Intriguingly, the identification of *Candida guilliermondii* as the culprit behind necrosis of the bowel segments deviates from the usual suspect, *Candida albicans*. *Candida guilliermondii*, which is part of the normal flora of human skin and mucosal surfaces, is involved in a range of infections, including chronic onychomycosis, acute osteomyelitis, septic arthritis, endocarditis, fungemia, and disseminated invasive infections [13]. It is one of the opportunistic fungi that recover most frequently in severely immunocompromised patients.

Our literature review confirmed that *C. guilliermondii* is a more common cause of candidemia in patients with cancer than in general hospital populations; however, it is rarely implicated in bloodstream infections occurring in other high-risk categories, such as intensive care unit patients [14]. Previously, the incidence of *C. guillermondii* in cancer patients appeared to be quite low. A review of 37 reports published between 1952 and 1992 revealed that *C. guilliermondii* was responsible for only 0.8% of all systemic Candida infections in this high-risk group [15]. The largest reported series included nine cases (two-thirds occurring in leukemia patients) observed over 11 years (1988–1998) at the M. D. Anderson Cancer Center [16]. However, recent studies of over 68 patients with over 79 episodes of infection with non-Candida species of *C. guillermondii* occurring in over 41% of patients with hematological malignancies showed that there has been an increase in the number of infections caused by non-Candida species from 3.6% (1998–2005) to 7.2% (2006–2013; *p*=0.0004). [4].

Initial diagnostic evaluations in this case were challenging because of the absence of culture and biopsy results, making it difficult to ascertain the etiology of intestinal necrosis. Upon receiving the biopsy, tissue smear, and deep wound culture results, a clearer picture emerged showing focal necrosis and stenosis, edema, microhematoma, and the presence of fungal organisms in vascular spaces and perivascular tissues, similar to findings in an autopsy case series [17–22] investigating gastrointestinal candidiasis where colonic involvement was identified in 20% of cases (22 out of 109). Among these, 82% (18 of 22) displayed multisite involvement in the gastrointestinal tract. The pathology report showed ulcers in 60% (15 of 25), plaque in 24% (6 of 25), erosion in 12% (3 of 25), and polyps in 4% (1 of 25) of cases.

References [17–22]. A comprehensive review of existing case reports indicates that all patients were immunocompromised, suffering from conditions such as malignancies, AIDS, end-stage renal disease, neutropenia, or diabetes mellitus, or were under immunosuppressive treatments.

Colonic candidiasis can manifest anywhere in the colon. Typical presenting symptoms included fever (71%, or 5 out of 7 cases), diarrhea (57%, or 4 out of 7 cases), abdominal pain (29%, or 2 out of 7 cases), and lower gastrointestinal bleeding (29%, or 2 out of 7 cases).

Dissemination, or widespread distribution of the disease, is a frequent occurrence, noted in 71% (5 out of 7) of cases, illustrating the invasive nature of the condition in immunocompromised patients.

Another concern is the resistance pattern of this fungus. Pfaller Madiekema Djmesser et al. [23, 24] recently assessed the recovery of rare Candida bloodstream isolates from various parts of the world, including 150 isolates of *C. guilliermondii*. The majority (85%–100%) was fully susceptible to amphotericin B, flucytosine, fluconazole, voriconazole, and ravuconazole, but susceptibility to itraconazole was much less common (10%). *C. guilliermondii* seems intrinsically resistant to echinocandins. High caspofungin MICs (>1 *μ*g/ml) have been reported for more than 95% of the tested isolates [25, 26], suggesting that this drug is unlikely to be effective against *C. guilliermondii* infections. A recent study showed that *C. guilliermondii* is also among the Candida species that are the least susceptible to echinocandin and anidulafungin [27]. Our data confirmed high rates of susceptibility to amphotericin B (100%), voriconazole (95%), fluconazole (90%), and flucytosine (86%); however, our isolates displayed lower rates of resistance to itraconazole (24%) and caspofungin (66%) than those observed in previous studies. The *Candida parapsilosis* complex (i.e., *C. parapsilosis*, *C. metapsilosis*, and *C. orthopsilosis*) and *Candida guilliermondii* are intrinsically less susceptible than other Candida species to echinocandins because of naturally occurring point mutations in the FKS gene [28–31]. A growing number of breakthrough infections with Candida species that have low susceptibility to echinocandins have been reported in patients receiving echinocandin therapy [32–40] This is also true in our case: despite being on the micafungin regimen, the patient developed necrosis and vascular bed invasion. Fortunately, in our case, the non-Candida fungus was susceptible to most of the antifungal therapy except for anidulafungin and was able to resolve the infection while on amphotericin B. There have been reports of single cases of *C. guilliermondii* infection displaying in vitro resistance to amphotericin B and/or fluconazole [41–44].

## 4. Conclusions

This case highlights the increasing incidence of systemic candidiasis in patients with hematological malignancies and the emergence of nonalbicans Candida species, such as Candida guilliermondii. Diagnostic challenges in early-stage intestinal necrosis emphasize the need for vigilant monitoring and prompt diagnostic investigations.

The observed resistance of *Candida guilliermondii* to echinocandins, necessitates careful selection of antifungal therapy. The patient's positive response to amphotericin B illustrates the importance of individualized treatment plans based on susceptibility patterns.

In conclusion, this case underscores the necessity for a comprehensive approach to managing *Candida guilliermondii* infections in immunocompromised patients. It reinforces the need for high suspicion of invasive fungal infections, tailored antifungal therapy, and continuous epidemiological surveillance. This case contributes valuable insights into resistance patterns and treatment responses, aiding in the optimization of treatment strategies and improvement of patient outcomes.

## Figures and Tables

**Table 1:** Susceptibility for our patient *Candida guillermondii fungus*.

	MDIL
*Anidulafungin*	1
*Micafungin*	0.5
*Caspofungin*	0.5
*5-fluorocytosine*	0.06
*Posaconazole*	0.25
*Voriconazole*	0.12
*Itraconazole*	0.5
*Fluconazole*	2
*Amphotericin B*	0.5

MDIL: minimum drug inhibitory level. The broth microdilution method was used to determine the patient's susceptibility.
